# Linking Oxidative Stress and Proteinopathy in Alzheimer’s Disease

**DOI:** 10.3390/antiox10081231

**Published:** 2021-07-30

**Authors:** Chanchal Sharma, Sang Ryong Kim

**Affiliations:** 1 School of Life Sciences, Kyungpook National University, Daegu 41566, Korea; chanchalmrt@gmail.com; 2 BK21 FOUR KNU Creative BioResearch Group, Kyungpook National University, Daegu 41566, Korea; 3 Brain Science and Engineering Institute, Kyungpook National University, Daegu 41944, Korea

**Keywords:** proteinopathy, reactive oxygen species, Alzheimer’s disease, amyloidopathy, tauopathy, oxidative stress

## Abstract

Proteinopathy and excessive production of reactive oxygen species (ROS), which are the principal features observed in the Alzheimer’s disease (AD) brain, contribute to neuronal toxicity. β-amyloid and tau are the primary proteins responsible for the proteinopathy (amyloidopathy and tauopathy, respectively) in AD, which depends on ROS production; these aggregates can also generate ROS. These mechanisms work in concert and reinforce each other to drive the pathology observed in the aging brain, which primarily involves oxidative stress (OS). This, in turn, triggers neurodegeneration due to the subsequent loss of synapses and neurons. Understanding these interactions may thus aid in the identification of potential neuroprotective therapies that could be clinically useful. Here, we review the role of β-amyloid and tau in the activation of ROS production. We then further discuss how free radicals can influence structural changes in key toxic intermediates and describe the putative mechanisms by which OS and oligomers cause neuronal death.

## 1. Introduction

**Figure 1:**
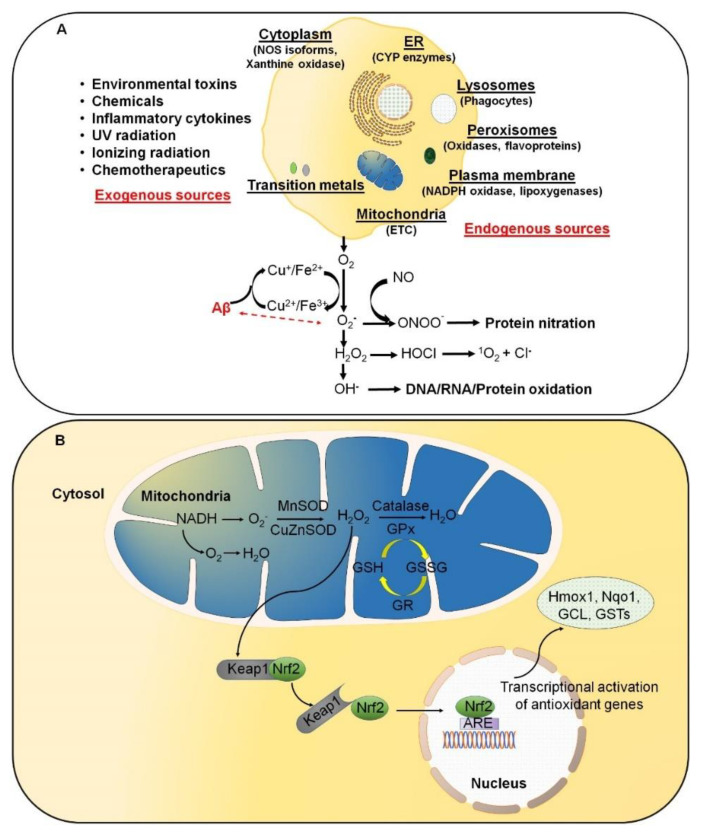
Excessive reactive oxygen species (ROS) are likely involved in the initiation and/or amplification of oxidative stress during the onset and progression of Alzheimer’s disease (AD). (**A**) ROS can be produced from both endogenous and exogenous sources. The endogenous sources of ROS include different cellular organelles, such as mitochondria, peroxisomes, and the endoplasmic reticulum, where oxygen consumption is high. (**B**) Under physiological conditions, a cellular balance is established between ROS generation and clearance, and is maintained by several antioxidative defense mechanisms.

Reactive oxygen species (ROS) result from normal daily cellular metabolism. Research conducted in the last two decades has clarified the role of ROS as secondary signaling molecules that regulate various biological and physiological processes, including proliferation, host defense, and gene expression [1,2]. Furthermore, earlier reports have also indicated the role of ROS as a signal transduction mechanism. This allows adaptation to changes in environmental nutrients and the oxidative environment [3]. In this respect, Kiley and Storz [4] have well defined, in the prokaryotes, mechanisms whereby ROS directly activates transcription factors (TFs) for stress adaptation. On the contrary, oxidative stress (OS) refers to elevated levels of intracellular ROS, such as superoxide anion (O_2_^•−^), hydroxyl radical (OH^•^), and non-radical molecules, such as hydrogen peroxide (H_2_O_2_) and singlet oxygen (^1^O_2_), which further damage lipids, proteins, and DNA (Figure 1A). A high-energy exposure or electron transfer reaction leads to the production of highly reactive ROS, which is a stepwise reduction of molecular oxygen (O_2_) as represented in equation (1). Moreover, ROS generation occurs at elevated rates in normal aging. It is an inevitable process in both acute and chronic pathophysiological conditions [5]. Thus, OS is usually the result of excessive ROS production, mitochondrial dysfunction, and an impaired antioxidant system, or a combination of these factors.

O_2_ → O_2_^•−^ → H_2_O_2_ → OH^•^ → H_2_O (1)

ROS are predominantly produced in mitochondria via mitochondrial enzymes. The electron transport chain (ETC) of mitochondria produces superoxide radicals at respiratory complexes I and III of the oxidative phosphorylation (OXPHOS) pathway through the single-electron leak [2,6]. Nevertheless, the rate of production of ROS in complex I is much less than the Flavin-dependent enzymes in the mitochondrial matrix [7]. Amongst various intracellular antioxidant enzymes, five have been mainly discussed in physiological conditions, i.e., (i) Cu/Zn-superoxide dismutase (Cu/Zn-SOD, SOD1) in the cytosol, (ii) manganese superoxide dismutase (Mn-SOD, SOD2) in the mitochondrial matrix, (iii) catalase (CAT), (iv) glutathione peroxidase (GPx), and (v) glutathione reductase. In Figure 1B, SOD converts superoxide to O_2_ and H_2_O_2_, whereas CAT and GPx convert H_2_O_2_ into H_2_O and O_2_. Along with the primary antioxidant defense against ROS, secondary antioxidant and cellular detoxification programs are mainly regulated by NF-E2-related factor 2 (Nrf2) and Kelch-like ECH-associated protein 1 (Keap1). Under normal conditions, Nrf2 is retained in the cytoplasm by the actin-binding protein Keap1; a substrate adaptor protein for the Cullin3-containing E3–ligase complex, which targets Nrf2 for ubiquitination and degradation by the proteasome [8]. Keap1 is redox sensitive since this protein can be modified by different oxidants and electrophiles [9]. OS abrogates the Keap1-mediated degradation of Nrf2, which in turn accumulates in the nucleus [10]. It heterodimerizes with a small musculoaponeurotic fibrosarcoma (Maf) protein on antioxidant response elements (AREs). Nrf2, along with ARE, further stimulates the expression of a wide array of phase II antioxidant enzymes, which includes NAD(P)H quinone oxidoreductase 1 (Nqo1), heme oxygenase 1 (Hmox1), glutamate-cysteine ligase, and glutathione S transferases (GSTs) [10,11,12]. In addition, Nrf2 also contributes to cellular proteostasis by regulating the expression of molecular chaperones and various proteasomal subunits [13,14,15]. Apart from antioxidant enzymes, small molecular weight and nonenzymatic antioxidants, such as vitamins, carotenoids, thiol antioxidants, and natural flavonoids, also protect intracellular components against ROS [16]. 

Deposition and spreading of aggregated proteins are the main characteristics of sporadic (s) and familial (f) forms of various neurodegenerative disorders, such as AD. This, in turn, results in excessive ROS production leading to OS, chronic neuroinflammation, and mitochondrial dysfunction, which altogether cause neuronal loss [17] and protein misfolding [18]. ROS-induced protein misfolding/unfolding can result in gain/loss-of-function. The protein modification of the oxidized proteins is insufficient to achieve their actual shape, impacting stability, activity, and/or function [19,20]. Several lines of evidence suggest that elevated ROS production initiates toxic amyloid beta precursor protein (APP) processing and thereby triggers amyloid-beta (Aβ) generation [21,22]. These elevations in ROS are the results of protein aggregation and corresponding neuronal damage, which in turn activates disease-associated microglia via damage-associated molecular patterns [23]. These ROS are primarily generated via NADPH oxidase 2, which is well associated with DAMP signaling, inflammation, and amyloid plaque deposition [23]. Additionally, ROS generated from mitochondria helps in the propagation of immune activation, leading to excessive OS and neurodegeneration. Interestingly, recent studies on postmortem AD brains and AD transgenic mice have shown that Aβ and APP are found in mitochondrial membranes to block protein transport and disrupt the ETC with final, irreversible cell damage [24]. Moreover, these disruptions are further exacerbated by a defective repair system. Tamagno and colleagues reported that OS resulting from hydroxynonenal (HNE) or H_2_O_2_ leads to enhanced Aβ production in different cell models [21]. In addition, HNE also modifies the γ-secretase substrate receptor nicastrin, which leads to enhanced binding of the γ-secretase substrate APP and likely results in elevated Aβ generation [22]. Moreover, neurons contain a high amount of polyunsaturated fatty acids (PUFAs) that can interact with ROS, leading to a self-propagating cascade of lipid peroxidation and molecular destruction [25]. Products of lipid peroxidation have also been shown to be elevated in blood samples and brains of AD patients at autopsy [26,27]. Both nuclear and mitochondrial DNA and RNA also exhibit oxidative damage in the AD brain [28,29,30]. Hence, understanding oxidative balance is regarded as an important event in understanding AD pathogenesis. OS might increase the aggregation and production of Aβ and assist polymerization and tau phosphorylation via the creation of a vicious cycle that stimulates the progression and even initiation of AD. Keeping this in mind, in this review, we sought to analyze the myriad interactions between oxygen radicals and toxic protein oligomers in the context of AD to understand their importance in disease pathogenesis. Furthermore, we also discuss the role of microbiota in altering redox balance and its consequences concerning Aβ production and tau hyperphosphorylation.

## 2. Markers of Oxidative Stress

ROS are oxygen-containing molecules that are more chemically reactive than O_2_ and, therefore, can damage cellular macromolecules. For example, ROS can react with nucleic acids (NA) by attacking nitrogenous bases and the sugar–phosphate backbone. Further, these can evoke single- and double-stranded DNA breaks, affecting the protein-coding region of mtDNA and influencing OXPHOS [31,32]. mtDNA mutations can cause disturbances in the respiratory chain, and as a result, it loses control over ROS production [1]. In addition, the modification in core DNA repair genes can result in an impaired recognition system and an inefficient repair of DNA damage, which in turn can accelerate aging and leads to age-related disruptions in cellular and tissue functions. This also results in the accumulation of ROS, which increases with age and intensifies OS. This elevation in OS damages mtDNA, leading to apoptosis, inhibition of mitochondrial respiratory chain transition, and increased mitochondrial membrane permeability in the absence of sufficient antioxidant capacity [5]. Thus, pro-oxidative/antioxidative cellular imbalance between ROS production and the ability of the defense mechanisms of biological systems to eliminate ROS-mediated cellular stress disturbances results in a vicious cycle, since the OS reciprocally aggravates ROS production. ROS have also been reported to attack structural and enzymatic proteins via oxidation of residual amino acids, prosthetic groups, formation of cross-links and protein aggregates, and proteolysis [32]. Lipid peroxidation (auto-oxidation) is a process in which PUFAs are oxidized due to several double bonds in their structure. This process involves producing peroxides (chemical compounds in which a single covalent bond links two oxygen atoms), ROS, and other reactive organic free radicals. Several markers of oxidative damage have been defined, including the following: 8-hydroxy-2-deoxyguanosine (8-OHdG) and 8-hydroxyguanosine, markers of oxidative DNA damage; 8-hydroxyguanine, a marker of RNA oxidation; protein carbonyls and nitrotyrosine, markers of protein oxidation; and malondialdehyde (MDA), thiobarbituric-acid-reactive substances, 4-hydroxy-2-nonenal (4-HNE), acrolein, isoprostanes, and neuroprostanes, markers of lipid peroxidation [5,32,33]. Moreover, ROS and aging have also been linked to the promotion and accumulation of advanced glycation end products (AGEs). AGEs are insoluble in detergents, protease-resistant, and non-degradable protein, lipid, or NA aggregates generated by non-enzymatic glycation or glycoxidation after exposure to aldose sugar. AGEs have been reported to impair normal cellular/tissue functions directly or indirectly through the AGE/RAGE pathway after binding to specific receptors for advanced glycation end products (RAGEs) [34]. Due to synergism with OS, the production of AGEs is promoted by OS, which eventually leads to ROS generation.

Furthermore, AGEs have been found to accumulate in numerous tissues throughout physiological aging, which leads to OS since the ability to respond to OS reduces with age. Due to this, many proteins lose their function, including those involved in gene transcription regulation [32,33]. Thus, AGEs serve not only as proinflammatory molecules but also as potent neurotoxins [35]. Protein glycation begins as a nonenzymatic process with a free amino acid group capable of producing a labile Schiff base. The process thus takes place along with the unconstrained condensation of aldehyde or ketone groups reportedly present in sugars. Furthermore, the phenomenon mentioned above also agrees with Maillard’s classical reaction in 1912 [36,37]. Subsequently, a series of reactions occur that result in the generation of AGEs containing irreversibly cross-linked heterogeneous protein aggregates. 

## 3. Linking OS and Proteinopathy in AD

The molecular associations of proteinopathy and proteotoxicity with OS are varied and complicated. Indeed, considerable evidence suggests that OS occurs before the appearance of symptoms in AD and that oxidative damage is detected not only in the vulnerable brain regions [38] but also in peripheral areas [36,38,39,40]. A reduction in the protein’s breakdown rate due to impaired proteasomal or lysosomal pathway or transcriptional activation or rapid translation of a specific mRNA may result in the accumulation of a specific hazardous protein [41,42,43]. In some circumstances, the mutant gene produces an abnormal protein product that is not cleared by the protein degradation machinery, causing it to accumulate. A similar event may occur in post-translationally modified proteins due to changes in the internal milieu of the cell, such as those observed in the redox status and kinase activity [42,43]. Excess accumulation of wild-type (WT) or mutant protein can precede various conformational alterations, e.g., helix to β-strand, facilitating oligomerization and self-aggregation. Heat shock proteins (HSP), such as HSP40, HSP70, HSP90, and other chaperones and co-chaperones, usually prevent misfolding of intracellular proteins. However, excessive accumulation, redox modifications, and/or mutations of such proteins may overwhelm this system and alter these chaperones’ expression [44,45]. The term proteotoxicity refers to the toxic effect of these protein aggregates on the various functions of cell organelles. ROS-responsive TFs can alter genes that encode such toxic proteins or enzymes involved in their production, processing, or degradation [46]. Furthermore, the proteasomal system, particularly the 26S proteasome, is responsible for degrading toxic protein aggregates and can be directly inactivated by OS to varying degrees. The in-depth mechanism of how ROS-mediated regulation of 26S proteasomal degradation occurs is currently being researched and clarified [47,48]. Furthermore, ROS may also influence the lysosomal clearance of toxic proteins, resulting in autophagy failure. The former is known to have an intricate relationship with autophagy, an intracellular degradation system [48]. ROS may also potentiate the oligomerization of proteins, such as Aβ, which interacts with transition metals (TM) such as iron (Fe), copper (Cu), zinc (Zn), etc., or other components capable of generating additional ROS (2–3). Likewise, proteotoxicity and mitochondrial dysfunction are also intertwined. Several studies conducted using isolated mitochondria, in vitro cell cultures, and postmortem brain samples showed that all forms of Aβ (monomer, oligomerized, or aggregated) cause a wide-ranging mitochondrial functional impairment which includes a reduction in bioenergetics, alteration in fusion/fission cycle, and impaired mitophagy [49,50]. Thus, proteotoxicity-induced mitochondrial dysfunction results in excess ROS production and triggers cell death pathways.

Aβ + TM → Aβ − TM(2)

Aβ − TM → TM − Aβ^+•^ (3)

### 3.1. Oxidative Stress and Aβ Proteinopathy

APP is a type I membrane protein that is synthesized and modified post-translationally in the endoplasmic reticulum (ER) and Golgi apparatus (GA) [51,52,53]. APP is further transported to the cell surface by a mechanism analogous to those used by other integral transmembrane proteins [51,52,53]. The metabolism of APP follows either a non-amyloidogenic pathway through α-secretase cleavage or an amyloidogenic pathway through cleavage mediated by β-site APP cleaving enzyme 1 (BACE1) [51,52,53]. Non-amyloidogenic processing predominantly occurs at the cell surface where α-secretase cleaves APP within the Aβ domain and generates a secreted large amino fragment ((s)APPα) and a small carboxyl (C)-terminal fragment (αCTF: C83) [51,52,53]. On the contrary, during the amyloidogenic processing, which takes place in the endosomes, BACE1 processes APP to a soluble β-cleaved ectodomain (sAPPβ) and a C-terminal fragment (βCTF: C99) [51,52,53]. This cleavage of APP results in the generation of toxic proteins termed Aβ peptides (Aβ42 and Aβ40 peptides), deposited as amyloid and neuritic plaques in extracellular brain regions [51,52,53]. Recent studies have demonstrated that APP is internalized through lipid rafts and clathrin-mediated endocytosis [53]. However, BACE1 is internalized by ADP ribosylation factor 6 endocytosis and is then sorted into early endosomes [53]. Further, the γ-secretase complex is responsible for the cleavage of βCTF (cleaved product of BACE1), which generates Aβ. Thus, generated Aβ is finally released into the extracellular space by fusing multi-vesicular bodies with the plasma membrane (PM) or is degraded via an endolysosomal pathway [52,53,54,55,56,57,58]. 

The expression, processing, and intracellular protein trafficking of APP and Aβ peptides reportedly occur in the trans-Golgi network, endosomes, and PM and are well-defined phenomena [57,58]. Importantly, endosomal changes, which are early events in AD progression, result in intra-neuronal Aβ accumulation and are correlated with redox imbalance, OS, synaptic dysfunction, cognitive impairment, and accelerated aging. During self-aggregation on neuronal membranes, a toxic aldehyde known as 4-HNE is produced, which causes lipid peroxidation and can cause ion-motive ATPases, glucose transporters, and glutamate transporters to malfunction [57,58,59]. In turn, Aβ promotes synaptic membrane depolarization, excessive calcium influx, and mitochondrial damage, impairing cells’ capacity to carry out normal physiological functions [60,61]. Thus, based on postmortem data and experimental studies carried out using cell lines, primary culture of hippocampal neurons, and transgenic animal models, it has been prominently suggested that Aβ peptide oligomers can interact with numerous astrocytic, microglial, and neuronal synaptic proteins, including α7- acetylcholine receptors (AChRs) and N-methyl-d-aspartate receptors (NMDARs); this, in turn, triggers a series of toxic synaptic events [58,59,60,61]. These events include abnormal activation of NMDARs (particularly NR2B-containing extrasynaptic NMDARs), increased neuronal calcium influx, calcium-dependent activation of calcineurin/PP2B, and its downstream signal transduction pathways involving cofilin, glycogen synthase kinase 2 beta (GSK-3β), cAMP response element-binding protein (CREB), and myocyte enhancer factor 2 (MEF2) [58,59,60,61]. This results in aberrant redox reactions and severing/depolymerizing F-actin, tau hyperphosphorylation, and endocytosis of α-amino-3-hydroxy-5-methyl-4-isoxazolepropionic acid receptors (AMPARs), which eventually leads to synaptic dysfunction and cognitive impairment and triggers the process of neurodegeneration in AD [56,57,58,59,60,61,62]. The inactivation of key proteins can lead to serious consequences in vital metabolic pathways. For instance, oxidized proteins can be harmful to membrane integrity. They may change the sensitivity of enzymes such as glutamine synthetase and creatine kinase, which are essential for brain function, to oxidative alterations [57,61,62]. This evidence suggests that Aβ trafficking pathways may be a therapeutic target through which disease manifestations may be improved [56,57,58,59,60,61,62]. 

Aβ toxicity has been demonstrated in vitro [63]. When placed in a physiological solution, Aβ precipitates into fibrils and generates free radicals. Casley et al. (2002) investigated the connection between Aβ and mitochondrial function using a cell-culture system [64]. They revealed that Aβ directly induces mitochondrial oxidative damage due to the generation of free radicals [64]. To this aim, they isolated rat mitochondria and incubated them with Aβ alone and with Aβ and nitric oxide (NO) together. They further measured the levels of tricarboxylic acid (TCA) enzyme complexes [65] and a-ketoglutarate dehydrogenase and pyruvate dehydrogenase activities [64]. Their findings revealed that Aβ significantly reduces mitochondrial respiration. Additionally, Aβ, together with NO, can further diminish mitochondrial respiration. In addition, they found that Aβ also inhibits the activities of cytochrome oxidase, a-ketoglutarate dehydrogenase, and pyruvate dehydrogenase [66,67]. Similarly, Kim et al. (2002) also showed that the addition of Aβ to isolated mitochondria from brain tissues taken directly from rats induces the release of cytochrome c and mitochondrial swelling [67]. These findings from the study by Kim et al. (2002) suggest that in AD, Aβ may accumulate intracellularly via abnormal APP processing. Its accumulation may exert neurotoxicity by interacting with mitochondria and causing oxidative damage apoptosis [67]. Furthermore, Tamagno et al. (2002) reported that the OS product 4-HNE could modulate BACE1. The NT2 neurons, when exposed to ascorbate/FeSO_4_ and H_2_O_2_/FeSO_4_, resulted in a significant generation of 4-HNE. They also reported that an increase in the levels of 4-HNE was well correlated with an increase in BACE1 protein levels and was accompanied by a proportional increase in carboxy-terminal fragments of APP [21,22]. They confirmed their findings by pretreating NT2 neurons with alpha-tocopherol, which is reported to prevent the formation of aldehydic end products of lipid peroxidation, including 4-HNE. These findings of Tamagno et al. (2002) support the hypothesis that OS and Aβ production are strictly interrelated events and that BACE1 inhibition may have a synergic therapeutic effect with antioxidant compounds [21,22]. 

In AD, the presence of elevated extracellular Aβ levels at potential sites of lipid peroxidation only serves to elevate the risk of oxidative damage. Compellingly, compared with age-matched controls, in areas such as the hippocampus, where AD pathology is concentrated, higher levels of 4-HNE in AD patients are observed [68]. Reports investigating patients with mild cognitive impairment confirm an increase in OS due to high levels of brain 4-HNE, an early event in AD pathogenesis [68,69,70]. Supporting this notion, OS markers, including lipid peroxidation, have been shown to precede and be accompanied by Aβ pathology in AD transgenic mouse models [71]. Recently, a study by Gwon and colleagues explained how OS could induce Aβ42 production via 4-HNE- or Fe^2+^-mediated modification of γ-secretase activity [72]. Using cultured human neuroblastoma (SH-SY5Y) cells and a luciferase reporter assay, they demonstrated that exogenous addition of 4-HNE or Fe^2+^ enhanced γ-secretase activity results in an increase in the Aβ42/Aβ40 ratio [72,73]. They further identified 4-HNE-mediated modification of nicastrin, a component of mature γ-secretase complexes, as the possible reason for the increase observed in the Aβ42/Aβ40 ratio. This could be because nicastrin liberates Aβ from APP, which may amplify amyloidogenic processing of APP via increased 4-HNE activation of γ-secretase activity [72,73]. However, the application of reduced glutathione (GSH) analog or the γ-secretase inhibitor (GSI) L685,458 could suppress the increase in γ-secretase activity [72,73]. Altogether, a positive feedback system might exist in which Aβ not only participates but also promotes lipid peroxidation, which in turn is facilitated by increases in extraneuronal Fe^2+^ [72,73].

Aβ has been reported to generate H_2_O_2_, a key ROS, from O_2_ through electron transfer interactions involving bound redox-active Cu^2+^ and Fe^3+^ [73,74]. H_2_O_2_ is readily converted into an aggressive OH radical by Fenton chemistry (4). These two types of ROS have been reported to be responsible for the early oxidative damage seen in AD. Some studies have shown that the levels of H_2_O_2_ generated by Aβ can be enhanced by co-incubation of the peptide with a reducing substrate, which becomes oxidized in the process [73,74,75]. For instance, using cholesterol as a reducing substrate, the resulting oxidation product will be 7β-hydroxycholesterol, proapoptotic and neurotoxic even at nanomolar concentrations. Thus, this molecule can also contribute to oxidative brain damage in AD [75].

H_2_O_2_ + Fe^2+^ → OH^•^ + HO^−^ + Fe^3+^ (4)

AGEs are regarded as chemical molecules that can be cross-linked to long-lived proteins [76,77]. In AD, enhanced oxidation of glycated proteins (i.e., glycoxidation) results in the extracellular accumulation of AGEs [78]. This has been confirmed in classic and primitive plaques observed in different cortical areas and senile plaques [78]. In vitro experiments conducted by Li and Dickson using double immunohistochemistry revealed AGE’s colocalization with apolipoprotein E (ApoE) [79]. They examined the binding of ApoE variants to AGE in the presence of bovine serum albumin and found that the dimeric form of ApoE has more binding specificity towards AGE. Furthermore, the results also suggested a three-fold higher binding activity between AGE and ApoE4 compared to binding activity between AGE and ApoE3, which signifies the pathogenic risk associated with ApoE4 in the case of fAD. AGE formation is reported to accelerate the formation of Aβ monomer to oligomeric forms [80]. Lines of evidence have shown prominent binding between Aβ and ApoE4, resulting in Aβ fibril formation and many subsequent pathways [79,80,81,82]. Recently, a cohort study involving the Dutch population revealed a higher association between AGEs and carriers of ApoE4 in progressive dementia [83].

Abnormal Cu, Zn, and Fe levels have been reported in the hippocampus and amygdala, along with severe histopathological changes in patients with AD [84,85,86]. Aβ generates ROS through different redox activities by binding to Cu or Fe (5–7) [74,86,87,88,89]. Cu^2+^ is reportedly found bound to several enzymes, such as SOD, cytochrome c oxidase, ceruloplasmin, and tyrosinase, which are involved in critical neuronal and non-neuronal cellular biochemical pathways. For instance, astrocytes can store and export Cu^2+^ to neurons. However, excess Cu^2+^ in astrocytes results in binding of Cu^2+^ to Aβ to form a cuproenzyme-like complex, which can transfer an electron to Cu^2+^ to convert Cu^2+^ to Cu^+^, thus forming the Aβ radical (Aβ^+•^) [74,85,86,87,88,89,90]. Furthermore, Cu^+^ can donate two electrons to O_2_ to generate H_2_O_2_ and produce OH radicals (Fenton-type reaction) [74,88,89]. Fe^2+^ is highly reactive, and excess of this metal ion often overproduces reactive chemical species (OH^•^) [74,91]. Fe accumulation is prominent in both in vitro and in vivo AD models that involve neuritic plaques, which further result in OS [92]. For instance, in SH-SY5Y cells overexpressing the Swedish mutant form of human APP, the intracellular Fe levels are significantly elevated along with increased OS [74]. The binding of Fe to Aβ results in the reduction of Fe^3+^ to Fe^2+^ and the generation of H_2_O_2_ [90,91]. As a critical component of amyloid plaques and cerebrovascular amyloidosis, Zn has also been reported to be involved in Aβ accumulation and ROS production in triple-transgenic mice [93]. It has been suggested that OH^•^ formation further damages biomolecules, such as lipids, proteins, and NA, due to the ability of OH^•^ to catalyze specific reactions, including hydrogen abstraction, addition reactions, and oxidation reactions in AD. These findings demonstrate that the interactions between Aβ and metals also produce ROS.

Fe^3+^/Cu^2+^ + O_2_^•−^ → Fe^2+^/Cu^+^ + O_2_Fe^3+^/Cu2^+^ + O_2_^•−^ → Fe^2+^/Cu^+^ + O_2_ (5)

Fe^2+^/Cu^+^ + H_2_O_2_ → Fe^3+^/Cu^2+^ + OH^•^ + OH-Fe^2+^/Cu^+^ + H_2_O_2_ → Fe^3+^/Cu^2+^ + OH^•^ + OH (6)

O_2_^•−^ → H_2_O_2_ → O_2_ + OH^•^ + OH-O_2_^•^ + H_2_O_2_ → O_2_ + OH^•^ + OH (7)

### 3.2. Oxidative Stress and Tau Proteinopathy

Besides Aβ proteinopathy, another prominent feature of AD pathogenesis is the accumulation of phosphorylated tau protein within neurons, known as neurofibrillary tangles (NFTs). These tau neurites also contribute to synaptic dysfunction and axonal degeneration [94]. Tau usually plays a significant role by stabilizing neuronal microtubules [95]. However, in AD, abnormal phosphorylation facilitates disassociation from the microtubule, resulting in the loss of function [96]. The change prompts self-assembly into highly toxic soluble oligomers, forming larger fibrils and tangles deposited within neurons [95,96]. Tau aggregates exhibit cell–cell transfer, which leads to seeding and further aggregation, supporting the concept of region to region spreading of phosphorylated tau in AD [97]. These plaques and NFTs are primarily deposited in brain regions, such as the hippocampus, amygdala, entorhinal cortex, and basal forebrain, which reportedly play an essential role in memory, learning, and emotional behaviors; plaques and NFTs reduce the number of synapses in these areas [55,98,99]. It has been suggested that an imbalance between kinases and phosphatases leads to aberrant tau hyperphosphorylation. As of now, nearly 28 protein kinases are known to be responsible for tau phosphorylation [100]. Furthermore, Aβ aggregates could be a component in a set of molecular events that lead to tau hyperphosphorylation [101,102]. For example, to favor the NFT formation, 4-HNE can induce alterations in tau protein structure which facilitates the participation of Aβ-induced OS in AD pathogenesis.

According to the published experiments and reports, OS is well associated with tau pathology. Moreover, cells with overexpressing tau protein are more vulnerable to the OS, likely caused by peroxisome depletion [103,104]. Tau protein can effectively induce ROS production in mitochondria. For instance, hippocampal tau phosphorylation in tau transgenic mice with the P301L mutation reportedly induces mitochondrial dysfunction, which results in H_2_O_2_ production, lipid peroxidation, and eventually neuronal loss [103,104,105,106]. Moreover, a reduction in cytoplasmic SOD1 or a deficit in mitochondrial SOD2 [107] increases tau phosphorylation in Tg2576 AD transgenic mice. Besides reducing nicotinamide adenine dinucleotide (NADH), ubiquinone oxidoreductase and mitochondrial dysfunction are also observed in the tau transgenic AD mouse model. This phenomenon has been well associated with elevated production of ROS, weakened synthesis of adenosine triphosphate (ATP), and mitochondrial respiration in aged animals [108]. 

Interestingly, P301S transgenic mouse brains showed enhanced OS and higher protein carbonyl levels in the cortical mitochondria. The relationship between tau pathology and OS was confirmed in P301L and P301S transgenic mouse models carrying the human tau gene with either the P301L or P301S mutation; these mice display accumulation of hyperphosphorylated tau, which causes neurodegeneration and the development of NFTs [109]. Tau, both directly and indirectly, influences mitochondrial function and mitochondrial transport along the neuronal axon, resulting in the reduction and impairment of mitochondria at presynaptic terminals with obvious deleterious consequences [110,111]. In AD-induced brains, phosphorylated tau was discovered to engage with voltage-dependent anion channel 1 (VDAC1), causing mitochondrial dysfunction [112]. As observed in AD postmortem brains and rodent models, tau hyperphosphorylation reduces complex I activity. It further causes a reduction in ATP generation, elevation in OS, mitochondrial membrane potential (mtΔΨ) dissipation, promotion of mitochondrial fission, and fragmentation [113]. Additionally, in a mouse model, mitochondrial stress was found to induce tau hyperphosphorylation [107]. These findings strongly suggest that tau pathology plays a significant role in mitochondrial dysfunction in AD.

Application of extracellular tau at different stages of aggregation to cortical co-cultures of neurons and astrocytes showed that only insoluble aggregates of tau could induce ROS production by activating nicotinamide adenine dinucleotide phosphate (NADPH) oxidase in a calcium-dependent manner [114]. The essential constituent of NFTs, the microtubule-associated protein tau (MAP-tau), was revealed to be vital to the formation of intracellular AGEs [115]. On the contrary, MAP-tau can be glycated in vitro, which decreases its capacity to bind to microtubules. Moreover, MAP-tau in the tubulin-binding region isolated from AD brains is glycated, leading to β-sheet fibril formation [116,117].

## 4. Linking Microbiota with Oxidative Stress and AD

Recently, several pieces of evidence link the role of microbiota in brain biology and aging, being an essential factor involved in various physiological processes via interactive symbiotic network system with host [118,119,120,121,122,123]. This interactive network between host and microbiota interconnects the gut track, epidermis, liver, and all other organs with the central nervous system, generally referred to as the microbiota–gut–brain axis [124,125]. The microbiota is composed mainly of bacteria that colonize all mucosal surfaces, with higher density in the gastrointestinal tract, approximately 100 trillion bacteria from nearly 1000 various bacterial species [118,124], thereby influencing and triggering various events associated with aging disorders such as AD [118,119,120,124,126]. Recently, a line of evidence revealed an association of brain amyloidosis with pro-inflammatory gut bacteria in cognitively impaired patients [127] and various AD mouse models [128,129]. These findings strongly highlight the association of microbiota and amyloid pathogenesis in AD. However, these fields lack crucial in-depth information and require more exploration. 

Physiological levels of OS have been generated in the microbiota, which can interfere with its composition and functionality [130]. Furthermore, interactions between microbe–microbe or host–microbiota may also impact the CNS redox balance by elevating ROS levels or impairing the antioxidant system or both [131,132]; hence, serving not only as a cause but also a consequence of increased levels of oxidative injury in CNS [131], thus adding a new dimension to the interplay between the gut microbiota and the brain. Moreover, the microbiota can also produce a considerable amount of CNS neurotransmitters, including dopamine, serotonin, and gamma-aminobutyric acid, that can modulate the local activity of the enteric nervous system and can correlate with their respective levels within the CNS, which in turn depends on the intestinal and BBB permeability [133]. The microbiota may also produce neurotoxic and potentially neurotoxic substances (such as lipopolysaccharides and amyloid proteins), which can also reach to CNS via the systemic circulation or the vagus nerve, promoting microglial activation and neuroinflammation, elevated ROS levels, and/or making neurons more susceptible to OS [133]. Therefore, gut microbes were considered plausible triggering factors for several neurodegenerative disorders, considering the proximity of enteric nervous system neurons to the intestinal lumen [134].

However, the production of amyloid proteins helps in the formation of bacterial biofilms by promoting the binding of bacterial cells with each other, thus providing resistance from physical or immune factor-mediated destruction [126]. However, in abnormal physiological conditions, bacterial amyloids may act as prion proteins and result in cross-seeding of amyloidogenic protein that elevates pathogenic Aβ formation both in vitro and in vivo [126,135,136,137,138]. For instance, the interaction of cyanobacteria with synaptic receptors such as NMDA results in upregulation of β-N-methylamino-l-alanine (BMAA), an OS-inducing neurotoxin [139,140], in AD brains. Furthermore, BMAA has been linked with protein misfolding and resulting inflammatory consequences in the AD mice model [139,140,141]. Numerous studies also suggested a link between activation of endogenous herpes simplex-1 (HSV-1) and amyloidogenesis in AD. This intimate relationship resulted in progressive neurodegeneration and cognitive impairment, contributing to AD pathogenesis [142,143,144]. A possible reason for this could be the alteration in gut dysbiosis, which results in increased gut barrier permeability, which in turn hyper activates the innate immune response that leads to systemic inflammation, thus impairing the blood–brain barrier [126], which results in neuronal injury, protein misfolding, and neurodegeneration leading to cognitive impairment [145]. In addition, overwhelmed microglial stimulation and NF-κB-mediated proinflammatory signaling and reactive oxidative and nitrosative stressors can result in neuronal and glial cell death, which can further impair phagocytosis, leading to the accumulation of Aβ42 [146,147]. C/EBPβ/AEP signaling was activated in 3xTg mice 5xFAD mice due to gut dysbiosis, resulting in Aβ aggregates, OS, and tau hyperphosphorylation [148]. 

Furthermore, reduction in the relative abundance of Proteobacteria and the low levels of Bifidobacteria can reduce beneficial short-chain fatty acids, leading to lipid peroxidation [149]. This, in turn, results in impaired APP processing and trafficking, thus impacting the production of Aβ. Studies conducted using germ-free mice have confirmed the impact of microbiota on microglia maturation, astrocyte activity, neuroinflammation, OS, protein misfolding, and cognitive impairment in AD pathogenesis [129]. Modifying the gut microbiota composition with food-based therapy or supplementing with probiotics may be helpful as a new preventive and therapeutic option in both in vitro and in vivo AD models and clinical trials [146,147,150,151,152,153,154].

## 5. Antioxidants and AD

It is now evident that Aβ and tau pathologies are modulated by ROS and are also self-perpetuating concerning ROS formation [155]. Hence, strategies involving inhibition of Aβ oligomerization or decreasing ROS production through the design of multitargeted compounds, such as antioxidants, have resulted in several promising approaches currently being tested in clinical trials. Antioxidants are a broad and heterogeneous collection of chemicals that work by inhibiting the production, detoxification, or scavenging of oxidant species. According to a different criterion, antioxidants can be classified into four different classes based on their chemical structure: vitamins (e.g., ascorbic acid, α-tocopherol, β-carotene, and retinol), synthetic compounds (e.g., butylated hydroxytoluene), natural compounds (e.g., plant-derived polyphenols), and inorganic compounds. Some antioxidants act as chain-breaking molecules, as they can prevent the propagation of or stop radical chain reactions (e.g., α-tocopherol). On the contrary, antioxidants, such as Gpx and catalase, can detoxify H_2_O_2_. This chemical reaction serves a vital role in cell biology as H_2_O_2_ can produce OH radicals in the presence of transition metals such as Fe^2+,^ for which there is no detoxification system [32].

Several antioxidant studies in AD models have also been reported, demonstrating that antioxidants consistently positively affect the animals’ behavioral and amyloidotic phenotypes (Table 1). Vitamins are potent antioxidants that directly affect free radicals by reducing OS, inflammatory processes, and neuronal loss [156]. Vitamin A (retinol) is essential for neuronal formation and remains present in the nervous system across life. Along with β-carotene, vitamin A also protects regenerating neurons during the neurodegeneration process by preventing the development and aggregation of Aβ plaques both ex vivo and in vivo. It may also prevent impaired cognition in AD and improve memory performance and spatial learning in rodent models. Studies have shown that AD patients have lower vitamin A and β-carotene compared with healthy individuals [156,157]. Early vitamin E (α-tocopherol) supplementation significantly reduced Aβ levels and deposition in the Tg2576 AD model [158]. The same therapeutic regimen prevents a surge in amyloidosis [158]. It improves cognitive function after experimental traumatic brain injury, a known risk factor for AD development in Tg2576 mice [159]. Curcumin, a popular antioxidant and anti-inflammatory substance found in curry spices, substantially decreases OS and amyloid pathology in the Tg2576 mouse model [160].

Furthermore, curcumin is a potent inhibitor of Aβ fibrillization [161] and oligomerization [162] and promotes destabilization of pre-existing Aβ deposits in both cell culture models and animal models of AD [160,161,162]. Curcumin and its derivatives also increase the uptake and clearance of Aβ by macrophages in AD patients [163]. Furthermore, using LLC-PK_1_ and NRK-52E cells, Balogun and colleagues reported that curcumin upregulates Aβ-induced SOD and catalase and can further activate Nrf2 by selectively binding to Keap1 [164]. Luteolin has also been associated with activating the Nrf2 pathway, which increases endogenous antioxidative gene expression in neuronal cells [165]. Melatonin, a drug with antioxidant properties, partially inhibits the expected time-dependent elevation in Aβ levels, reduces the abnormal nitration of proteins, and increases the survival of Tg2576 mice [166]. Similarly, ferulic acid, rosmarinic acid, and nordihydroguaiaretic acid (NDGA) have also been reported to inhibit the fibrillization and/or oligomerization of Aβ into higher-order species in vitro [167,168,169,170].

The significant outcomes of these studies are reductions in Aβ levels, phosphorylated tau, mitochondrial dysfunction, microglial activation, enhanced synaptic activity, and amelioration of cognitive decline. These results indicate that antioxidant treatment is beneficial in reducing and/or preventing AD progression. The findings also show that combination therapy positively impacts cognitive behavior and lowers AD pathology. The positive findings of these studies are promising. However, they warrant prospective studies (e.g., antioxidant treatment of elderly individuals without AD) and clinical trials (antioxidant treatment for patients with AD). Recent work has also highlighted the importance of a healthy and detoxified innate response by consuming diet precursors and enhancing responsiveness [171]. For instance, the application of radiation health, such as UV radiation from the Sun, can prepare an individual for further UV exposure [172]. Another example includes exposure to pro-oxidants such as H_2_O_2_, which can prepare the body for subsequent pro-oxidant exposure, which is similar to the formation of antibodies in vaccines. 

## 6. Conclusions

ROS are the byproducts of normal cell metabolism and are therefore unavoidable. However, an imbalance between pro-oxidative and antioxidative cellular mechanisms leads to a vicious cycle since OS reciprocally aggravates ROS production, which results in the oxidation of lipids, proteins, and NA in neurons. This oxidation is a frequently encountered pathological marker in the case of AD. It contributes to the disease’s progression by increasing Aβ deposition, hyperphosphorylation of tau, and synaptic and neuronal loss. Altogether, the relationship between OS and AD suggests that OS is an essential part of the pathological process and that antioxidants may be helpful in treating AD. However, AD demands a precisely targeted treatment. Furthermore, non-antioxidant, targeted protection against OS, including transition metal chelators, compounds that modify the oligomeric structure, and inhibitors of enzymatic ROS production (such an NADPH oxidase), may potentially exert a strong therapeutic effect against AD. Additionally, consumption of precursors in the diet and mild exposure to pro-oxidants can benefit future exposure to the same stressor. All of these approaches are currently being rapidly developed. 

## Figures and Tables

**Table 1:** Overview of the experimentally documented roles of various known natural antioxidant compounds in cases of Alzheimer’s disease.

Antioxidant	Mechanism	Experimental Model	Reference
Vitamins			
α-Tocopherol	Reduces Aβ and lipid peroxidation; delays development of tau pathology; reduction in learning deficits and motor weakness	Tg2576 mice	[156,157,167,168,169,170,173]
Ascorbic acid	Reduces Aβ oligomers, tau phosphorylation, and oxidative stress	hAPP-J20 mice	[174]
Retinol	Reduces MDA levels; upregulates SOD activity; reduces Aβ pathology	APPswe/ PS1M146V/ tauP301L (3 × Tg) mice; in vitro enzymatic assay and in silico modeling	[175]
Naturally present			
CoQ10	Reduces MDA levels; upregulates SOD activity; reduces Aβ pathology	Tg19959 mice; APP/PS1 Tg mice	[176,177]
Synthetic			
Mito Q	Prevents cognitive decline, oxidative stress, Aβ accumulation, synaptic loss, and caspase activation	3 × Tg mice	[178]
Plant-based			
Zeolite	Increases endogenous SOD; reduces Aβ levels and plaque burden	Randomized clinical trial	[179]
β-Carotene	Improves cognitive impairment and oxidative stress	Streptozotocin-induced AD mice model	[180]
Curcumin	Inhibits Aβ fibrillization and oligomerization; clearance of Aβ by macrophages; reduces Aβ40 and 42 and Aβ-derived diffusible ligands; increases Aβ-degrading enzymes; promotes destabilization	Tg2576 mice; APPSw mice; APPswe/PS1dE9 mice; in vitro enzymatic assay	[160,161,162,163,181]
Ferulic acid	Inhibits the fibrillization and/or oligomerization of Aβ	In vitro enzymatic assay	[167,168]
Rosmarinic acid	Inhibits the fibrillization and/or oligomerization of Aβ	Molecular docking analysis; Tg2576 mice; PC12 neuroblastoma	[168,169,170]
Nordihydroguaiaretic acid (NDGA)	Inhibits the fibrillization and/or oligomerization of Aβ	Tg2576 mice	[168]
Mimetic			
ApoE mimetic peptide Ac-hE18A-NH2	Reduces oxidative stress and ApoE secretion; inhibits Aβ plaque deposition	APP/PS1ΔE9 mice and U251 human astrocyte cells	[182]
Catalase mimetic	Protects against oxidative stress, DNA, and protein oxidation; reduces Aβ and tau phosphorylation	3 × Tg-AD mice	[183]
Drug			
Melatonin	Inhibits time-dependent elevation of Aβ; reduces abnormal oxidation and nitration of proteins; increases survival; alleviates learning and memory deficits; decreases choline acetyltransferase activity and increases acetyltransferase activity; increases mitochondrial function	Tg2576 mice; APP 695 Tg mouse model; APP/PS1 mice; APPswePS1dE9 mice	[166,184,185,186,187]
N-Acetyl-l-cysteine	Reduces lipid peroxidation, oxidative stress, and glutathione peroxidase activity	APP/PS-1 knock-in mice	[188]

## Abbreviations

ROS: Reactive oxygen species; AD: Alzheimer’s disease; OS: Oxidative stress; TFs: Transcription factors; ETC: Electron transport chain; OXPHOS: Oxidative phosphorylation; Nrf2: NF-E2-related factor 2; Keap1: Kelch-like ECH-associated protein 1; Maf: Musculoaponeurotic fibrosarcoma; AREs: Antioxidant response elements; Nqo1: NAD(P)H quinone oxidoreductae 1; Hmox1: Heme oxygenase 1; GSTs: Glutathione S transferases; APP: Amyloid beta precursor protein; Aβ: Amyloid beta; HNE: hydroxynonenal; PUFAs: Polyunsaturated fatty acids; NA: Nucleic acids; 8-OHdG: 8-hydroxy-2-deoxyguanosine; MDA: Malondialdehyde; 4-HNE: 4-hydroxy-2-nonenal; AGEs: Advanced glycation end products; RAGEs: Receptors for AGEs; WT: Wild-type; HSP: Heat shock proteins; TM: Transition metals; ER: Endoplasmic reticulum; GA: Golgi apparatus; BACE1: β-site APP cleaving enzyme 1; PM: Plasma membrane; AChRs: α7-acetylcholine receptors; NMDARs: N-methyl-d-aspartate receptors; GSK-3β: Glycogen synthase kinase 3 beta; CREB: cAMP response element-binding protein; MEF2: Myocyte enhancer factor 2; AMPARs: α-Amino-3-hydroxy-5-methyl-4-isoxazolepropionic acid receptors; NO: Nitric oxide; TCA: Tricarboxylic acid; ApoE: Apolipoprotein E; NFTs: Neurofibrillary tangles; NADH: Nicotinamide adenine dinucleotide; ATP: Adenosine triphosphate; VDAC1: Voltage dependent anion channel 1; mtΔΨ: Mitochondrial membrane potential; NADPH: Nicotinamide adenine dinucleotide phosphate; MAP: Microtubule-associated protein; NDGA: Nordihydroguaiaretic acid.
